# Ischemia augments alloimmune injury through IL-6-driven CD4^+^ alloreactivity

**DOI:** 10.1038/s41598-018-20858-4

**Published:** 2018-02-06

**Authors:** Mayuko Uehara, Zhabiz Solhjou, Naima Banouni, Vivek Kasinath, Ye Xiaqun, Li Dai, Osman Yilmam, Mine Yilmaz, Takaharu Ichimura, Paolo Fiorina, Paulo N. Martins, Shunsuke Ohori, Indira Guleria, Omar H. Maarouf, Stefan G. Tullius, Martina M. McGrath, Reza Abdi

**Affiliations:** 1 Transplantation Research Center, Renal Division, Brigham and Women’s Hospital, Harvard Medical School, Boston, MA USA; 2 Renal Division, Brigham and Women’s Hospital, Harvard Medical School, Boston, MA USA; 3 Division of Nephrology, Boston Children Hospital, Harvard Medical School, Boston, MA USA; 4 Division of Surgery, University of Massachusetts Medical School, Boston, MA USA; 5 Division of Transplant Surgery and Transplantation Surgery Research Laboratory, Brigham and Women’s Hospital, Harvard Medical School, Boston, MA USA

## Abstract

Ischemia reperfusion injuries (IRI) are unavoidable in solid organ transplantation. IRI augments alloimmunity but the mechanisms involved are poorly understood. Herein, we examined the effect of IRI on antigen specific alloimmunity. We demonstrate that ischemia promotes alloimmune activation, leading to more severe histological features of rejection, and increased CD4^+^ and CD8^+^ T cell graft infiltration, with a predominantly CD8^+^ IFNγ^+^ infiltrate. This process is dependent on the presence of alloreactive CD4^+^ T cells, where depletion prevented infiltration of ischemic grafts by CD8^+^ IFNγ^+^ T cells. IL-6 is a known driver of ischemia-induced rejection. Herein, depletion of donor antigen-presenting cells reduced ischemia-induced CD8^+^ IFNγ^+^ allograft infiltration, and improved allograft outcomes. Following prolonged ischemia, accelerated rejection was observed despite treatment with CTLA4Ig, indicating that T cell costimulatory blockade failed to overcome the immune activating effect of IRI. However, despite severe ischemic injury, treatment with anti-IL-6 and CTLA4Ig blocked IRI-induced alloimmune injury and markedly improved allograft survival. We describe a novel pathway where IRI activates innate immunity, leading to upregulation of antigen specific alloimmunity, resulting in chronic allograft injury. Based on these findings, we describe a clinically relevant treatment strategy to overcome the deleterious effect of IRI, and provide superior long-term allograft outcomes.

## Introduction

Ischemia reperfusion injury (IRI) is an inevitable consequence of transplantation. IRI leads to a cascade of intra-graft inflammation, and initiates immune activation within the transplanted organ^1^. Controlling innate immunity early on post-transplantation is a key component of innovative strategies to promote allograft acceptance^2,3^. Furthermore, critical organ shortages have necessitated the increased use of organs from donors of older age or with co-morbid diseases for transplantation^4–6^. Owing to pre-existing damage, these organs have shorter expected duration of function and more readily accumulate ischemic injuries, which can further compromise their long-term outcomes^7–13^. Therefore, an improved understanding of the link between IRI and increased allograft immunogenicity has highly practical applications for the field of transplantation.

Antigen presenting cells (APC) within the allograft are activated by danger signals released during IRI^14,15^. In particular, allograft-resident dendritic cells (DC) are perfectly poised to regulate the interplay between innate and antigen-specific alloimmunity^16–19^. We have previously shown that allograft-resident DCs increase IL-6 production in the setting of ischemia, and blockade of IL-6 improves allograft outcomes^18^. IL-6 plays a key role in alloimmune injury both by directly increasing alloimmune responses and indirectly, by augmenting inflammation and innate immunity, which also promote graft rejection^20–23^. However, the effector mechanism linking IL-6, allospecific T cell activation and chronic rejection has not been identified.

Here, we used both a typical Class I MHC mismatch model, and an antigen-specific TCR transgenic model of cardiac transplantation to comprehensively examine the impact of IRI on antigen-specific alloreactive CD4^+^ and CD8^+^ T cells. OTI transgenic mice express a transgenic CD8^+^ T cell receptor, and OTII transgenic mice express a transgenic CD4^+^ T cell receptor, both of which are reactive to OVA. Transgenic mice expressing OVA on all cells were used as donors in our studies^24,25^. Using the OVA/OT system, we transplanted ischemic and control OVA hearts into OTI and OTII recipients, and studied the subsequent activation of alloreactive CD4^+^ T cells.

In this study, we observed that IRI is associated with accelerated allograft rejection, characterized by allograft infiltration with CD8^+^ IFNγ^+^ T cells. We identified allospecific CD4^+^ T cells as critical mediators of enhanced alloimmune reactivity following IRI. However, despite their central role, costimulatory blockade of CD4^+^ T cells with CTLA4Ig failed to overcome the negative effect of prolonged ischemia on allograft survival. Addition of anti-IL-6 therapy to CTLA4Ig overcame the effect of severe allograft ischemia, leading to long-term graft survival in a full MHC mismatch model. This approach represents a clinically applicable treatment model to reduce early immune activation by IRI and improve long-term allograft outcomes.

## Results

### Ischemia augments alloimmunity

**Figure 1:**
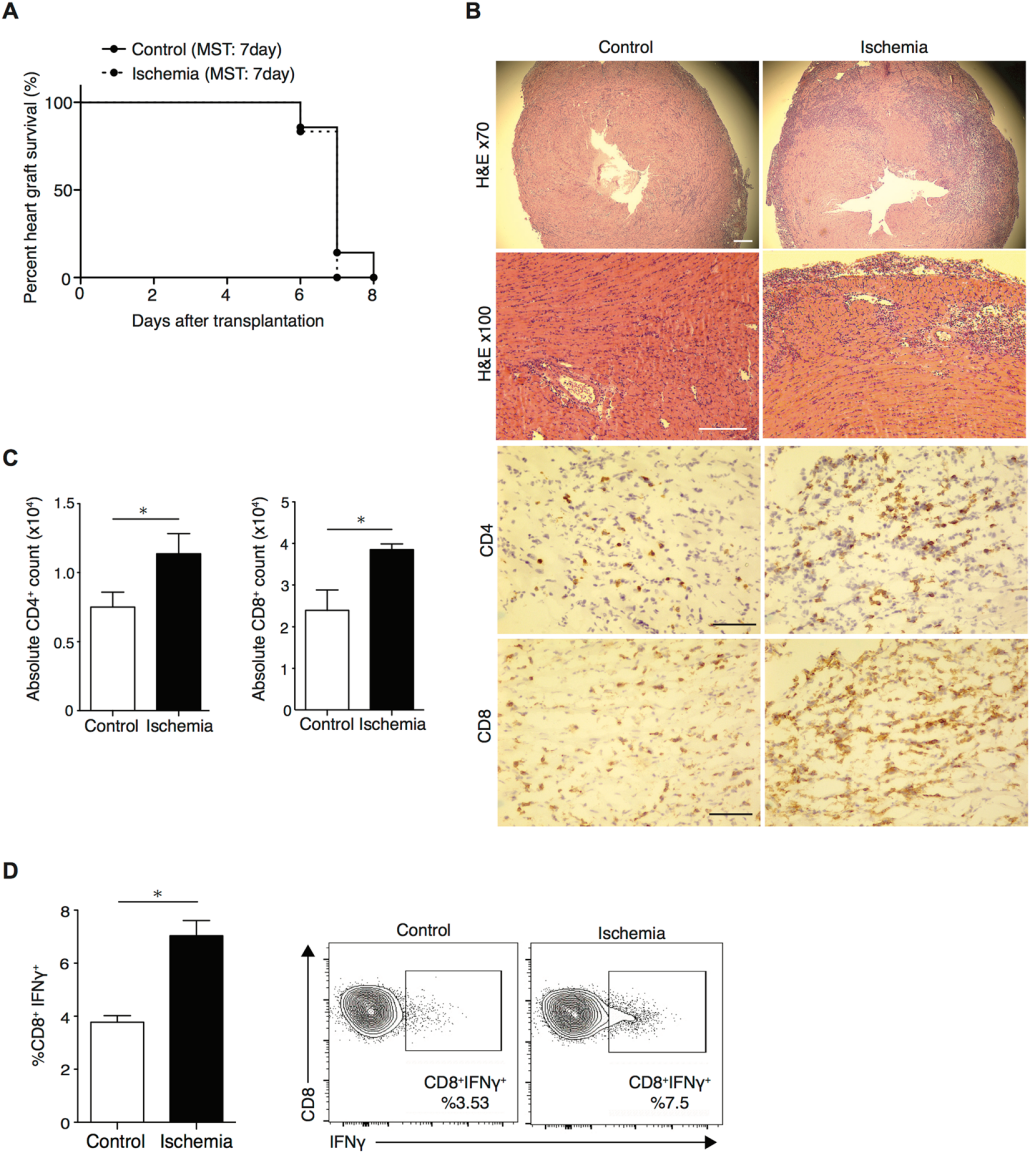
Ischemia is associated with increased graft infiltration and augmentation of the alloimmune response in a complete allo-mismatch model. Full MHC mismatch BALB/c hearts were harvested and transplanted into C57BL/6 recipients within 30 minutes (control group) or after 8 hours stored in UW solution, at 4 °C (ischemic group). Recipients were followed for transplant survival or grafts were harvested at day 6. (**A**) No difference was observed in tempo of graft rejection between control and ischemic group (MST: 7 vs. 7 days, *p* = 0.3, n = 6–7/group) (**B**) Histological analysis at 6 days post-transplant showed increased tissue injury and increased CD4^+^ and CD8^+^ T cell infiltration of ischemic heart grafts as compared to controls. (Scale bar 100 μm for H&E stain, 50 μm for CD4 and CD8 stain). (**C**) Enumeration of absolute number of cardiac graft-infiltrating cells, as assessed by flow cytometry of control and ischemic grafts, revealed a greater number of CD4^+^ and CD8^+^ infiltrating ischemic grafts (7.5 × 10^3^ ± 1 × 10^3^ vs. 12.6 × 10^3^ ± 1.2 × 10^3^, *p < 0.05 for CD4^+^ T cells and 24 × 10^3^ ± 4.8 × 10^3^ vs. 38.5 × 10^3^ ± 1.4 × 10^3^, *p < 0.05 for CD8^+^ T cells, respectively, n = 3/group). (**D**) A marked increase in the frequency of IFNγ-producing CD8^+^ T cell in ischemic grafts as compared to controls (7.0 ± 0.5% vs. 3.7 ± 0.2%, *p < 0.05, respectively, n = 3/group). Representative flow plots, gating on CD8^+^ T cells.

BALB/c hearts were harvested and transplanted into fully MHC mismatched C57BL/6 recipients within 30 minutes (control group) or after storage at 4 degrees Celsius, immersed in University of Wisconsin (UW) solution, for 8 hours (ischemic group). Recipients were followed for transplant survival. We observed no change in graft survival between groups (MST: 7 vs.7 days, n = 6–7 mice/group, *p* = 0.3) (Fig. 1A). However, ischemic grafts harvested at day 6 post-transplant showed increased cellular infiltration with CD4^+^ and CD8^+^ T cells compared to control, as assessed by histological analysis (Fig. 1B). Graft infiltrating cells were isolated on day 6 post-transplant from control and ischemic grafts and analyzed by flow cytometry. Absolute cell numbers were enumerated and were significantly higher for both CD4^+^ and CD8^+^ T cells in ischemic vs. control grafts. (12.6 × 10^3^ ± 1.2 × 10^3^ vs. 7.5 × 10^3^ ± 1 × 10^3^, *p < 0.05 for CD4^+^ T cells and 38.5 × 10^3^ ± 1.4 × 10^3^ vs. 24 × 10^3^ ± 4.8 × 10^3^, *p < 0.05 for CD8^+^ T cells, respectively, n = 3/group) (Fig. 1C). Furthermore, the percentage of IFNγ producing CD8^+^ T cells was significantly higher in ischemic grafts compared to controls (7.0 ± 0.5% vs. 3.7 ± 0.2%, *p < 0.05, respectively, n = 3/group) (Fig. 1D).

### Allospecific CD8^+^ T cells lead to prompt rejection, which is not augmented by IRI

The relative importance of antigen specific CD4^+^ and CD8^+^ T cells in alloimmune responses induced by IRI is unknown. Given the predominance of CD8^+^ IFNγ^+^ T cells in rejecting ischemic allografts in the full MHC mismatch model, we initially focused on antigen specific CD8^+^ T cells. To assess this, we transplanted control and ischemic hearts from OVA donors into CD8^+^ TCR transgenic OTI recipients. Grafts in both groups were rapidly rejected with no difference in survival between ischemic and control hearts (MST: 3 vs. 3 days, *p* = 0.51, n = 5/group) (Supplementary Figure 1A). Both groups showed similar severity of cellular infiltration and tissue necrosis (4 days post-transplant) suggesting that in a strongly alloreactive CD8^+^-driven model of rejection, IRI does not augment the CD8^+^ response (Supplementary Figure 1B).

### CD4^+^ alloreactive T cells play a critical role in augmenting alloimmune responses by IRI

**Figure 2:**
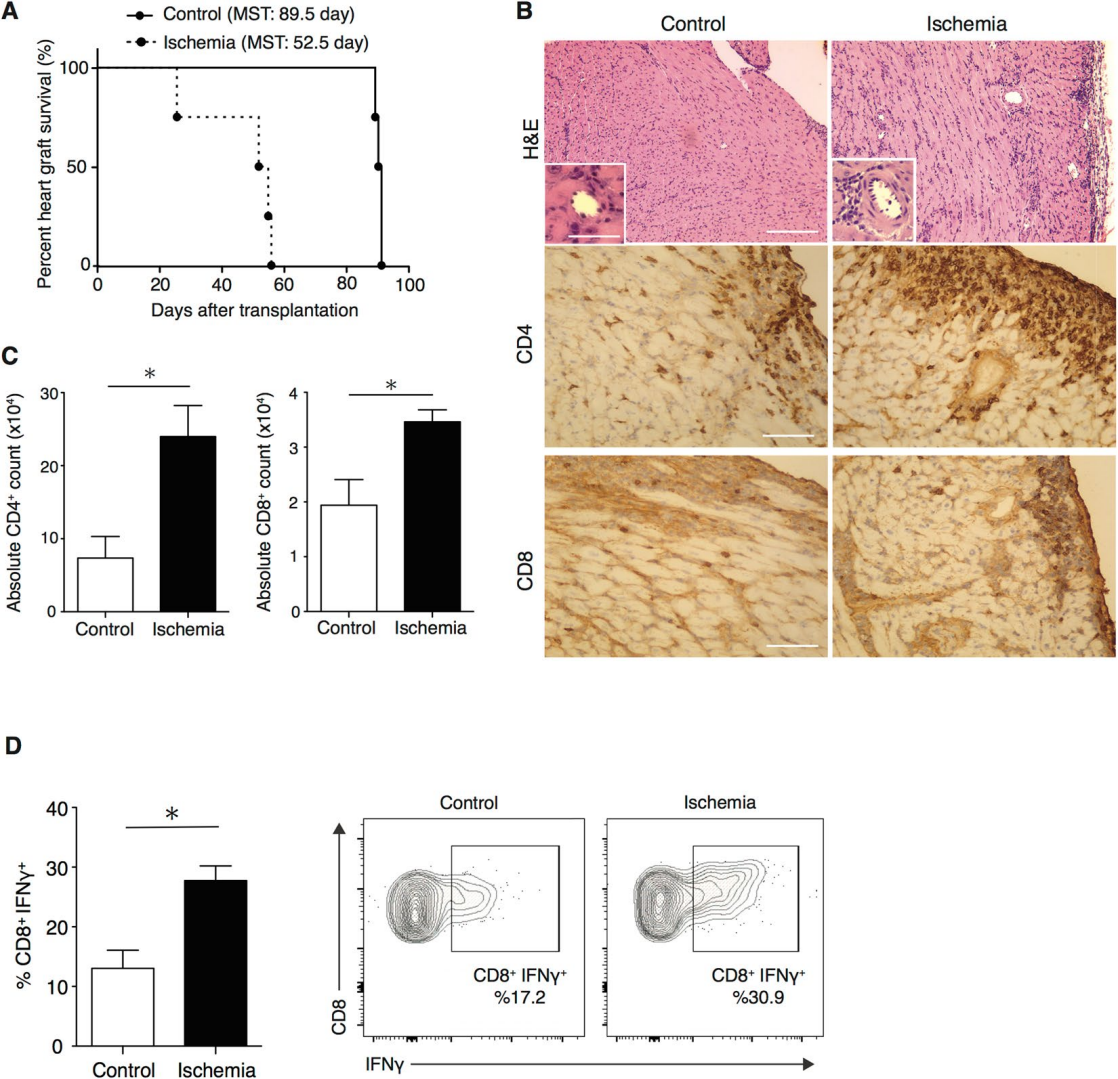
Ischemia promotes accelerated rejection in a CD4^+^ allospecific model of transplantation and is associated with increased graft-infiltrating CD8^+^ IFNγ producing T cells. Control and ischemic OVA hearts were transplanted into OTII recipients. (**A**) Ischemic OVA heart grafts showed accelerated rejection as compared to controls (MST: 52.5 vs. 89.5 days, **p < 0.01, n = 4/group). (**B**) Histology of ischemic OVA heart grafts showed an increase in tissue necrosis, inflammatory cell infiltrates and an increase in intra-graft CD4^+^ and CD8^+^ T cells on day 6 post-transplant compared to controls. (Scale bar 100 μm for H&E, CD4 and CD8 stain. Inset scale bar 30 μm). (**C**) Quantification of graft-infiltrating CD4^+^ and CD8^+^ T cells by flow cytometry shows a significantly greater number of CD4^+^ and CD8^+^ T cells in ischemic grafts compared to control (23.9 × 10^4^ ± 4.2 × 10^4^ vs 7.3 × 10^4^ ± 2.9 × 10^4^, *p < 0.05 for CD4^+^ T cells and 3.4 × 10^4^ ± 0.2 × 10^4^ vs 1.9 × 10^4^ ± 0.4 × 10^4^, *p < 0.05 for CD8^+^ T cells, respectively, n = 3/group). (**D**) A significantly greater number and percentage of IFNγ-producing CD8^+^ T cells in ischemic grafts as compared to controls, (27.77 ± 2.46% vs. 13.04 ± 3.04%, *p < 0.05, n = 3/group). Representative flow plots, gated on CD8^+^ T cells.

To examine the role of antigen specific CD4^+^ T cells in allograft rejection augmented by IRI, control and ischemic OVA hearts were transplanted into CD4^+^ TCR transgenic OTII hosts. Ischemic grafts underwent accelerated allograft rejection as compared to control (MST: 52.5 vs. 89.5 days, **p < 0.01, n = 4/group) (Fig. 2A). Histologically, ischemic grafts showed greater tissue destruction and more severe infiltration by CD4^+^ and CD8^+^ T cells at day 6 post-transplant (Fig. 2B). To quantify infiltrating T cells, graft-infiltrating lymphocytes were isolated at day 6 post-transplant and analyzed by flow cytometry. The absolute numbers of CD4^+^ T cells were 7.3 × 10^4^ ± 2.9 × 10^4^ vs. 23.9 × 10^4^ ± 4.2 × 10^4^ and CD8^+^ T cells were 1.9 × 10^4^ ± 0.4 × 10^4^ vs. 3.4 × 10^4^ ± 0.2 × 10^4^ in control and ischemic groups, respectively (*p < 0.05, n = 3/group) (Fig. 2C).

### CD8^+^ IFNγ^+^ T cells are the predominant intra-graft lymphocyte population in ischemia induced alloreactivity

To assess the intra-graft immune response, we next characterized graft infiltrating T cell populations in OT II recipients of OVA hearts. Control and ischemic grafts were harvested at day 6 post-transplant; graft-infiltrating lymphocytes were isolated and analyzed by intracellular cytokine staining and flow cytometry. Ischemic allografts had similar numbers of Foxp3^+^ regulatory T cells (absolute cell count: 6498 ± 844.7 vs. 5364 ± 2925.1, *p* = 0.6, n = 3/group), but significantly greater numbers of CD4^+^ IL17^+^ (absolute cell count: 2615 ± 461.5 vs. 816.1 ± 328.9, *p < 0.05, n = 3/group) and CD4^+^ IFNγ^+^ (absolute cell count: 8518 ± 1503 vs. 2206 ± 888.8, *p < 0.05, n = 3/group) than control grafts (Supplementary Figure 2A,B and C, respectively), indicating that IRI promotes pro-inflammatory allospecific CD4^+^ T cell infiltration in this heart transplant model. Similar to the full mismatch transplant model, we observed a marked increase in IFNγ producing CD8^+^ T cells at 6 days post-transplant in ischemic grafts as compared to controls (27.77 ± 2.46% vs. 13.04 ± 3.04%, *p < 0.05, respectively, n = 3/group) (Fig. 2D), suggesting that in the setting of IRI, CD4^+^ alloreactive T cells mediate alloimmune injury via IFNγ producing CD8^+^ T cells.

Finally, to assess peripheral alloimmune responses, spleen and draining lymph nodes (DLN) of OT II recipients of control or ischemic OVA hearts were harvested at day 6 post-transplant and analyzed by flow cytometry. No differences were seen in the frequency of CD4^+^ Foxp3^+^ regulatory T cells (Tregs), CD4^+^ IL17^+^ and CD8^+^ IFNγ^+^ producing T cells in the spleen or graft DLN (Supplementary Figure 3A and B for spleen and DLN, respectively). However, recipients of ischemic heart grafts showed greater allospecific IFNγ production by splenocytes as assessed by ELISPOT analysis (68.17 ± 3.32 vs. 25.63 ± 3.45 spots/10^6^ cells, ***p < 0.001, respectively, n = 3/group) (Supplementary Figure 4Α). These findings indicate that accelerated alloimmune injury induced by IRI in the early post-transplant period is driven largely by local, intra-graft alloimmune activation in this model.

### Depletion of CD4^+^ alloreactive T cells abrogates augmentation of intra-graft IFNγ producing CD8^+^ T cells

**Figure 3:**
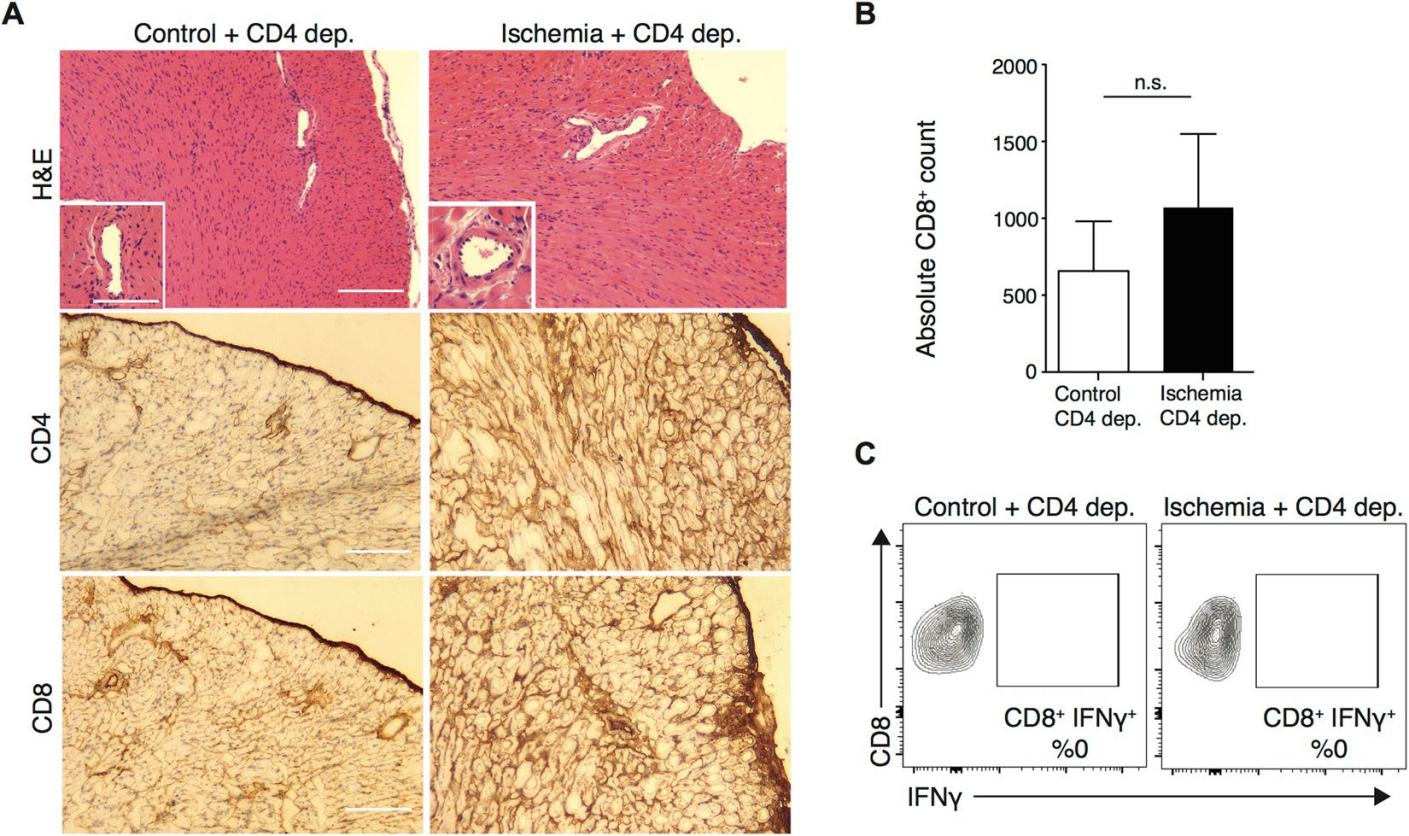
Depletion of CD4^+^ alloreactive T cells abrogates IRI-induced augmentation of intra-graft IFNγ-producing CD8^+^ T cells. OTII recipients were treated with anti-CD4 antibody prior to receiving control or ischemic OVA hearts. Grafts were harvested at day 6 post-transplant for analysis. (**A**) No differences in allograft infiltration or evidence of rejection seen in either group following CD4 depletion. No intra-graft CD4^+^ T cells were detected and rare CD8^+^ T cells were detected by histological analysis, with no notable difference between control and ischemic organs. (Scale bar 100 μm for H&E, CD4 and CD8 stain. Inset scale bar 30 μm). (**B**) There was no statistical difference between absolute number of CD8^+^ T cells infiltrating control and ischemic grafts as enumerated by flow cytometry (657 ± 322 vs. 1069 ± 478, *p* = 0.5, n = 3/group). (**C**) No IFNγ-producing CD8^+^ T cells were detected in the grafts after CD4^+^ depletion (n = 3/group).

To further examine the importance of allospecific CD4^+^ T cells in IRI induced alloimmunity, we depleted CD4^+^ T cells from OTII recipients using anti-CD4 antibody, administered on day −3 to −1 prior to transplantation. Depletion was confirmed by peripheral blood flow cytometry. Following CD4^+^ depletion, OVA control and ischemic heart allografts were transplanted into OTII recipients. Histological analysis of grafts harvested at day 6 revealed healthy myocytes without evidence of inflammation in either control or ischemic allografts. Immunohistochemical staining and lymphocyte enumeration by flow cytometry showed a marked reduction in the number of CD8^+^ T cells in both control and ischemic heart grafts at day 6 post-transplant (657 ± 322 vs. 1069 ± 478, *p* = 0.5, respectively) (Fig. 3A and B). Graft-infiltrating CD8^+^ IFNγ^+^ T cells were also rare in both control and ischemic grafts following CD4^+^ depletion (Fig. 3C) (compare to Fig. 2D). ELISPOT analysis of splenocytes harvested on day 6 showed that CD4^+^ T cell depletion led to a marked reduction in the frequency of allospecific IFNγ producing splenocytes in both groups, with no difference observed between recipients of control and ischemic heart grafts (2.4 ± 0.5 vs. 9.3 ± 3.7 spots/10^6^ cells, *p* = 0.13, respectively, n = 3/group). (Supplementary Figure 4B).

### Graft resident APC promote alloreactive CD4^+^ T cells infiltration following IRI

Cardiac antigen presenting cells (APC) comprise a mixed population of DCs, macrophages and monocytes^26–28^ and act as a critical link between innate and adaptive alloimmune responses. We have previously reported that ischemia promotes autophagy and increased IL-6 production by ischemic allograft resident DCs^18^. To examine the role of graft-resident APC in promoting CD8^+^ IFNγ^+^ T cell infiltration following IRI, we depleted donor cardiac APC by administration of liposomal clodronate to the donor prior to transplantation, as previously described^29^. Following clodronate treatment, we saw significant depletion of CD11c^+^ DCs in heart grafts, as assessed by flow cytometry at the time of transplantation (control vs. clodronate treatment; 109.9 × 10^3^ ± 13.0 × 10^3^ vs. 29.2 × 10^3^ ± 8.4 × 10^3^, **p < 0.01) (Supplementary Figure 5A). A non-significant decrease in intra-graft F4/80^+^ macrophages was also observed. (control vs. clodronate treatment; 545.1 × 10^3^ ± 223.5 × 10^3^ vs. 318.1 × 10^3^ ± 74.7 × 10^3^, *p* = 0.39) (Supplementary Figure 5B).

**Figure 4:**
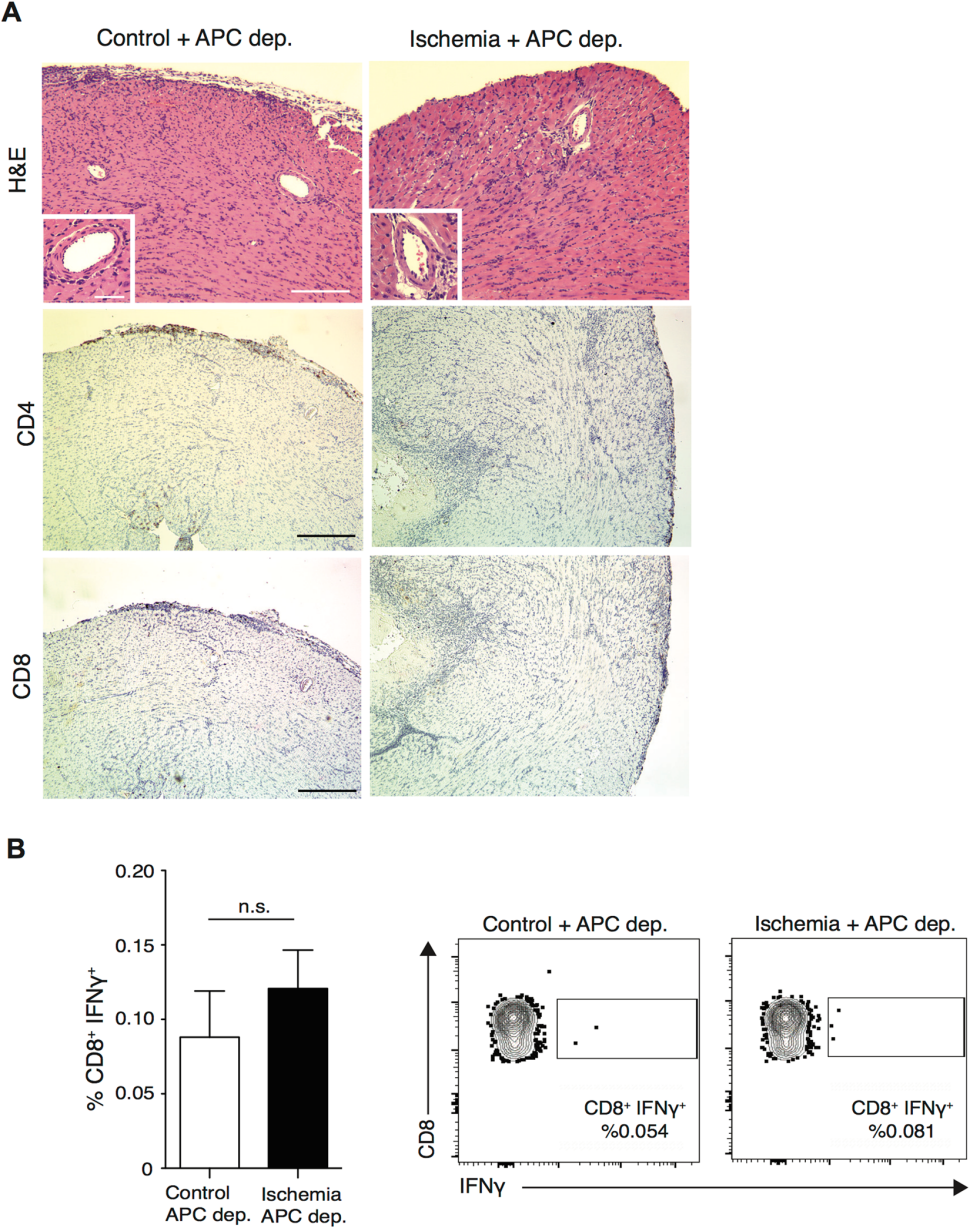
Intra-graft APCs play major role in recruiting alloreactive T cells to ischemic heart allografts. OVA heart donors were depleted of APCs by I.P. administration of liposomal clodronate on day −8, −5 and −1 prior to transplantation. Control and ischemic hearts from clodronate treated-OVA donors were transplanted in to OTII recipients and grafts were harvested at day 6 post-transplant. (**A**) Histology of APC-depleted heart grafts showed mild inflammatory cell infiltrates, clear patent vasculature, with similar pattern between control and ischemic grafts. Infiltration of CD4^+^ and CD8^+^ T cells were similar between control and ischemic grafts. (Scale bar 100 μm for H&E, CD4 and CD8 stain. Inset scale bar 30 μm). (**B**) APC depleted control and ischemic heart grafts showed minimal infiltration with IFNγ producing CD8^+^ T cells, similar in both groups (0.09 ± 0.03% vs. 0.12 ± 0.02%, *p* = 0.46, n = 3/group); Representative flow plots, gated on CD8^+^ T cells.

APC-depleted control and ischemic OVA grafts were harvested on day 6 post-transplant. Both groups showed a similar degree of cellular infiltration and tissue injury with very few graft-infiltrating CD4^+^ and CD8^+^ T cells observed by histological analysis (Fig. 4A). Similarly, no differences were seen in CD8^+^ IFNγ^+^ T cell allograft infiltration of control versus ischemic grafts (0.09 ± 0.03% vs. 0.12 ± 0.02%, control vs. ischemia, *p* = 0.46) (Fig. 4B). Of note, APC depletion led to a highly significant reduction in frequency of CD8^+^ IFNγ^+^ T cells in ischemic grafts as compared to non-depleted ischemic grafts (compare to Fig. 2D) (0.12 ± 0.02% vs. 27.7 ± 2.46%, ***p < 0.001). These data indicate that depletion of donor intra-graft APC prior to transplantation abrogated the enhanced alloreactive T cell infiltration observed after IRI.

### Early production of IL-6 by APC as a mechanism to promote alloreactive CD4^+^ T cell infiltration following IRI

We have previously shown that ischemic DCs produce a large amount of IL-6 *in vitro*, a key inflammatory cytokine potentiating the activity of alloreactive T cells^18,30,31^. We next sought to assess its role in the augmented alloimmune response observed after IRI. Control and ischemic OVA hearts were transplanted into OTII recipients and mRNA was extracted from hearts harvested on day 1, 3, and 6 post-transplant. Comparing ischemic with control grafts, we found that the relative expression of IL-6 in ischemic heart allografts was significantly elevated. It peaked at day 1 post-transplant (*p < 0.05, n = 4/group), and remained significantly elevated up to day 3 (*p < 0.05, n = 3/group) (Supplementary Figure 6A).

To investigate the source of this early increase in IL-6, control and ischemic OVA heart grafts were harvested at day 1 post-transplant into OTII recipients. At this early time point, very few graft infiltrating CD4^+^ or CD8^+^ T cells were present (Supplementary Figure 6B). We therefore hypothesized that ischemic APC, including allograft resident DCs, are the major source of IL-6 in the graft early post-transplant. Indeed, ischemic and control allografts harvested from clodronate-treated donors had a significant reduction of IL-6 expression, with undetectable IL-6 levels at day 1 post-transplant in both groups after APC depletion (n = 3/group) (Supplementary Figure 6C).

### Antagonizing IL-6 markedly reduces allograft infiltration by T cells following IRI

To test the functional importance of IL-6 in IRI induced alloimmunity, OTII recipients of control and ischemic OVA hearts were treated with anti-IL-6 0.1 mg by intraperitoneal injection on days 0 to 3 and on day 5 post-transplant. On day 6 post-transplant, grafts were harvested and analyzed for infiltrating T cells.

**Figure 5:**
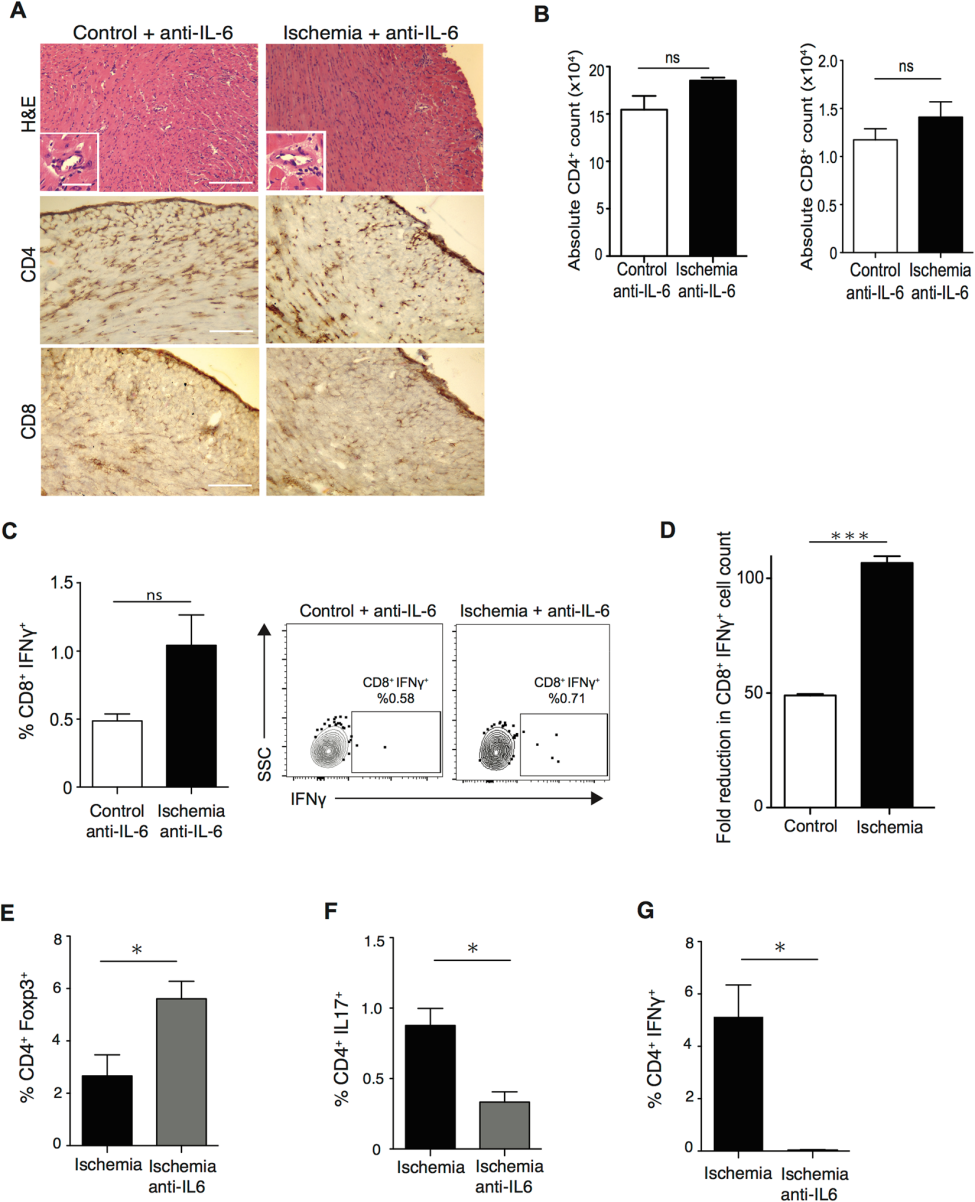
Antagonizing IL-6 markedly reduces alloreactive T cell infiltration following IRI. (**A**) Control and ischemic OVA hearts were transplanted into OTII recipients. Recipients were treated with anti-IL-6 and heart grafts were harvested at day 6. No difference was noted in cell infiltration, CD4^+^ and CD8^+^ T cell infiltration between control and ischemic grafts. (Scale bar 100 μm for H&E, CD4 and CD8 stain. Inset scale bar 30 μm). (**B**) Absolute number of CD4^+^ and CD8^+^ T cell by flow cytometry analysis were similar in both groups (15.4 × 10^4^ ± 1.4 × 10^4^ vs. 18.5 × 10^4^ ± 0.1 × 10^4^, *p* = 0.1 for CD4^+^ T cells and 1.1 × 10^4^ ± 0.1 × 10^4^ vs. 1.4 × 10^4^ ± 0.1 × 10^4^, *p* = 0.29 for CD8^+^ T cells, respectively, n = 3/group). (**C**) Minimal CD8^+^ IFNγ^+^ T cell infiltration was observed and no statistical difference was seen between ischemic and control allografts (0.48 ± 0.05% vs. 1.04 ± 0.2%, *p* = 0.05, n = 3/group). Representative flow plots, gated on CD8^+^ T cells. (**D**) Marked reduction in graft infiltration by CD8^+^ IFNγ^+^ T cells seen following anti-IL-6, with greater effect in ischemic organs. (Fold reduction in CD8^+^ IFNγ^+^: control vs. ischemia 49.04 ± 0.52 vs. 106.74 ± 2.97, ***p < 0.001). (**E**) Significantly higher percentage of CD4^+^ Foxp3^+^ T cells was seen in anti-IL-6 treated ischemic graft compared to untreated ischemic graft (5.61 ± 0.67% vs. 2.66 ± 0.81%, *p < 0.05, n = 3/group). (**F**) Significantly lower percentage of CD4^+^ IL17^+^ T cell was seen in anti-IL-6 treated ischemic graft compared to untreated ischemic graft (0.33 ± 0.07% vs. 0.88 ± 0.12%, *p < 0.05, n = 3/group). (**G**) Significantly lower percentage of CD4^+^ IFNγ^+^ T cell was seen in anti-IL-6 treated ischemic graft compared to untreated ischemic graft (0.04 ± 0.02% vs. 5.13 ± 1.21%, *p < 0.05, n = 3/group).

IL-6 blockade prevented IRI-induced allograft infiltration, with an overall reduction in intra-graft CD4^+^ and CD8^+^ T cells, (Fig. 5A). Following anti-IL-6, no differences were observed in the number of T cells in control versus ischemic grafts (15.4 × 10^4^ ± 1.4 × 10^4^ vs. 18.5 × 10^4^ ± 0.1 × 10^4^, *p* = 0.1 for CD4^+^ T cells and 1.1 × 10^4^ ± 0.1 × 10^4^ vs. 1.4 × 10^4^ ± 0.1 × 10^4^, *p* = 0.29 for CD8^+^ T cells, respectively, n = 3/group) as assessed by flow cytometry of graft-infiltrating leukocytes (Fig. 5B). Furthermore, treatment with anti-IL-6 significantly decreased CD4^+^ and CD8^+^ T cell infiltration of ischemic grafts (anti-IL-6 treated ischemic grafts vs. untreated ischemic controls: 18.5 × 10^4^ ± 0.1 × 10^4^ vs. 27.2 × 10^4^ ± 2.6 × 10^4^, *p < 0.05 for CD4^+^ T cells and 1.41 × 10^4^ ± 0.1 × 10^4^ vs. 3.4 × 10^4^ ± 0.2 × 10^4^, ***p < 0.001 for CD8^+^ T cells, respectively, n = 3/group) (Fig. 5B: ischemia + anti-IL-6 and Fig. 2C: ischemia).

Anti-IL-6 led to a decrease CD8^+^ IFNγ^+^ T cell infiltration in both groups, resulting in similar frequencies in treated control and ischemic allografts (0.48 ± 0.05% vs.1.04 ± 0.2%, *p* = 0.05, respectively, n = 3/group) (Fig. 5C). To specifically assess the effect of anti-IL-6 on IRI-induced CD8^+^ IFNγ^+^ T cell infiltration, we compared treated versus untreated grafts in both control and ischemic groups. There was a notable reduction in frequency of CD8^+^ IFNγ^+^ T cells in both groups following anti-IL-6, but the effect size was markedly greater in ischemic grafts (49.04 ± 0.52 vs. 106.74 ± 2.97 fold reduction in CD8^+^ IFNγ^+^ infiltration, control vs. ischemia, ***p < 0.001, respectively, n = 3/group) (Fig. 5D). These data indicate that IL-6 blockade overcame the immune stimulating effect of IRI and prevented IRI-induced allograft infiltration by CD8^+^ IFNγ^+^ T cells.

As IL-6 is intimately involved in CD4^+^ T cell differentiation, we also examined the effect of anti-IL-6 on other T cell populations. OVA hearts were harvested and stored for 8 hours in ischemic conditions. OT II recipient mice were treated with anti-IL-6 versus control. On Day 6 post-transplant, heart grafts were harvested and analyzed by flow cytometry. Anti-IL-6 led to a significant increase in the frequency of allograft infiltrating Tregs in treated ischemic grafts as compared to untreated ischemic controls (5.61 ± 0.67% vs. 2.66 ± 0.81%, *p < 0.05, n = 3/group) (Fig. 5E). Furthermore, treatment with anti-IL-6 led to a significantly lower percentage of CD4^+^ IL17^+^ T cells (0.33 ± 0.07% vs. 0.88 ± 0.12%, *p < 0.05, n = 3/group) (Fig. 5F) and CD4^+^ IFNγ^+^ T cells (0.04 ± 0.02% vs. 5.13 ± 1.21%, *p < 0.05, n = 3/group) (Fig. 5G) in ischemic hearts.

### Anti-IL-6 synergizes with CTLA4Ig even under severe IRI conditions

We next moved to a more clinically applicable treatment model comprising transplantation across full MHC mismatch (BALB/c hearts into C57BL/6 recipients), and more severe IRI conditions. This experimental design is more consistent with real-world clinical scenarios. Prior to transplantation, BALB/c heart grafts were stored for 16 hours (severely ischemic) versus transplanted within 30 minutes (control). To assess the efficacy of IL-6 blockade in this model, recipient mice were treated with anti-IL-6 (0.1 mg by intraperitoneal injection on days 0 to 3 and on every other day until Day13) and observed for heart graft survival. In this full MHC mismatch model, anti-IL-6 therapy alone failed to prolong heart graft survival in either control or severely ischemic hearts (MST: untreated control vs. anti-IL-6 treated control: 7 vs. 7 days, *p* = 0.12, untreated ischemia vs. anti-IL-6 treated ischemia: 7 vs. 7 days, *p* = 0.47, respectively, n = 3–6/group) (unchanged from untreated survival, see Fig. 1A).

**Figure 6:**
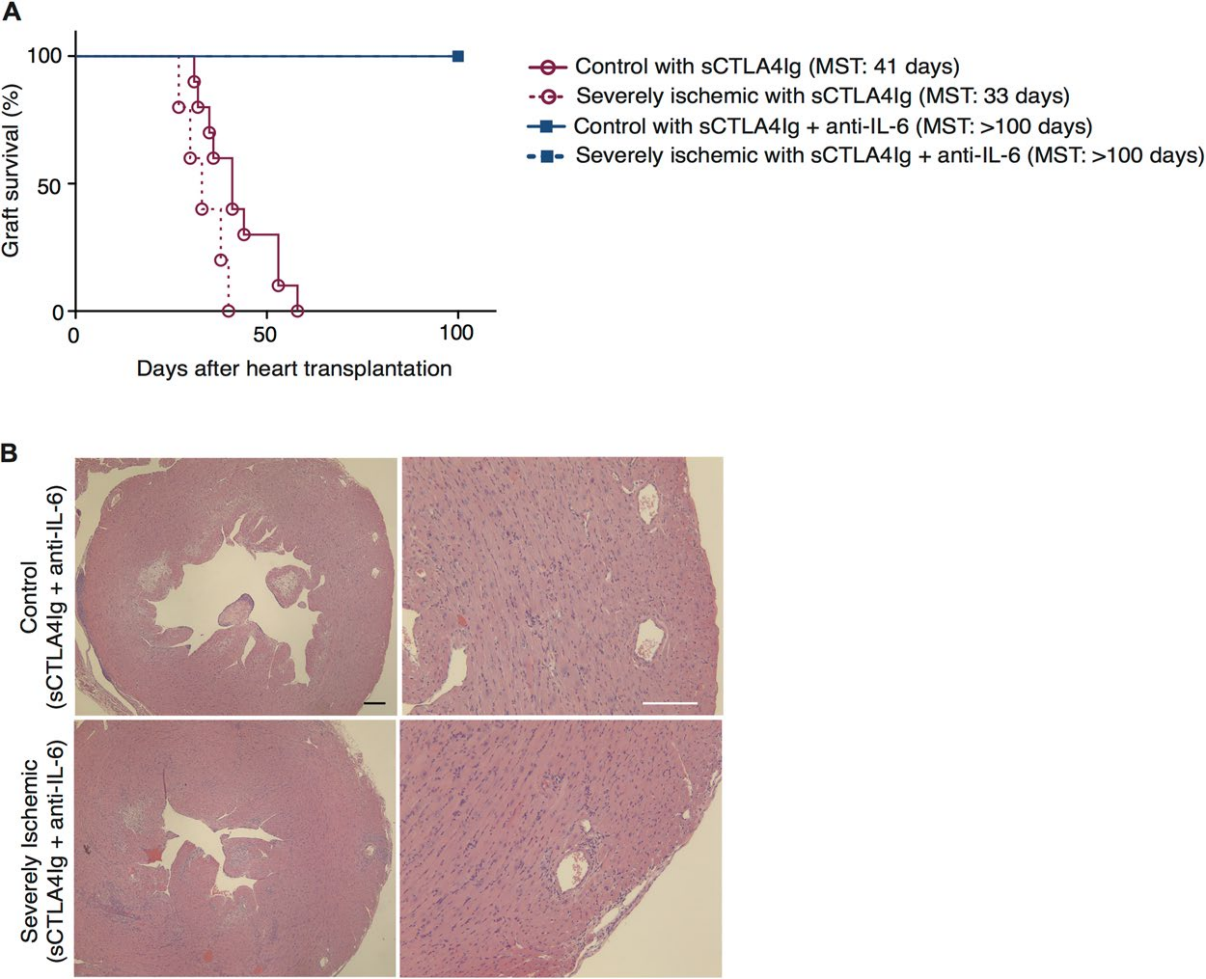
Anti-IL-6 synergizes with CTLA4Ig to improve graft outcomes, even under severe IRI conditions. BALB/c hearts were harvested and transplanted into full MHC mismatched C57BL/6 recipients within 30 minutes (control) or after 16 hours (severely ischemic). All recipients were treated with single dose of CTLA4Ig on day 2 (sCTLA4Ig). Anti-IL-6 was given as described, until day 13. (**A**) Despite treatment with sCTLA4Ig, severely ischemic grafts underwent accelerated rejection compared to control. Addition of anti-IL-6 to sCTLA4Ig treatment overcame the effect of severe ischemia, and both control and severely ischemic grafts survived for more than 100 days. (**B**) Histology at 100 days post-transplant of control and severely ischemic BALB/c heart grafts into C57BL/6 recipients and treated with sCTLA4Ig and anti-IL-6. Both control and severely ischemic grafts showed only minimal lymphocyte infiltration and little vasculopathy. (Scale bar 100 μm).

We next assessed if T cell costimulation blockade alone could overcome the immune-activating effect of severe IRI. Allograft recipients were treated with a single dose of CTLA4Ig on day 2 after transplantation (250 μg IP: sCTLA4Ig). Following sCTLA4Ig, severely ischemic allografts had significantly accelerated allograft rejection compared to sCTLA4Ig-treated non-ischemic control (MST: 33 vs. 41 days, *p < 0.05, respectively, n = 5–10/group) (Fig. 6A).

To directly target the effect of severe ischemia, recipients were treated with anti-IL-6 and sCTLA4Ig, and observed for heart graft survival. Surprisingly, both control and severely ischemic allografts treated with sCTLA4Ig and anti-IL-6 had long-term graft survival (MST: > 100 vs. > 100 days, respectively, n = 3/group) (Fig. 6A). Grafts were harvested at day 100 and assessed for evidence of chronic allograft rejection. Following treatment with both agents, histological analysis of severely ischemic grafts demonstrated similar lymphocyte infiltration and vasculopathy compared to control non-ischemic grafts (Fig. 6B), indicating that anti-IL-6 synergized with sCTLA4Ig and overcame the effect of severe ischemia on alloimmune activation to produce long term graft survival.

## Discussion

Early intra-organ inflammation plays a pivotal role in augmenting alloimmunity^32–34^. IRI is an inevitable consequence of transplantation and numerous studies have shown that IRI plays a central pathogenic role in initiating inflammatory responses and is a major determinant of graft survival^2,35,36^. One of the key unmet needs in the field of transplantation is to better understand the mechanisms involved, and devise targeted therapies to oppose this effect. Strategies to reduce the long-term impact of IRI are of greater clinical significance given the increasing reliance on organs from older donors or those with comorbid disease, which are more susceptible to these injuries^11–13^.

IRI leads to intra-graft inflammation and innate immune activation through multiple pathways. An exaggerated reperfusion response is seen following partial liver transplantation, with enhanced macrophage infiltration and upregulation of pro-inflammatory cytokines, leading to poorer outcomes^37^. Following ischemia, antibody binding to Fc receptors expressed on innate immune cells is increased, leading to accelerated transplant vasculopathy, irrespective of antibody specificity^38^. In lung transplantation, IRI promotes graft-infiltrating neutrophils which interact with graft-resident DCs, resulting in increased MHC Class II expression, IL-12 production and expansion of IFNγ producing CD4^+^ and CD8^+^ T cells^39^. Most recently, several lines of evidence suggest that IRI leads to complement activation via the mannose-binding lectin pathway^40,41^. Indeed, blockade of complement activation using a C1 inhibitor prevented IRI-induced accelerated allograft rejection in a murine cardiac transplant model^40,41^.

Although the cellular mechanisms involved in alloimmune activation following IRI have been investigated polyclonally, alloantigen specific models allow a more in-depth examination of increased cellular immunity after IRI^42–45^. Using an antigen-specific model, we have performed a detailed analysis of the increased cellular immunity following IRI. Data from transplantation of OVA donor hearts into OTI or OTII recipients reveal the critical importance of CD4^+^ T cells in promoting the accumulation of intra-graft CD8^+^ IFNγ producing cells. CD4^+^ T cell depletion markedly reduced the number of IFNγ producing CD8^+^ T cells within the ischemic allograft following IRI. This finding is in keeping with prior studies demonstrating that in the absence of CD4^+^ T cells, CD8^+^ T cells alone could not mediate cardiac allograft rejection^46^.

Prior work has demonstrated the presence of a population of endogenous memory CD8^+^ T cells in naïve mice, which were observed infiltrating the graft within 48 hours of reperfusion and cause accelerated rejection following prolonged cold ischemia^42,47^. These early-infiltrating endogenous CD8^+^ T cells show little dependence on CD4^+^ T cell help, and in contrast to our model, CD4^+^ T cell depletion led to only a modest increase in allograft survival^42^. This difference suggests that these are two different populations; in contrast to endogenous memory CD8^+^ T cells, CD8^+^ graft infiltrating T cells observed on day 6 may be primary donor-reactive CD8^+^ T cells, which require CD4^+^ T cell help to develop into effector T cells. Interestingly, in both models, allograft infiltrating CD8^+^ IFNγ^+^ T cells induce rejection that is resistant to CTLA4Ig. Our studies were focused primarily on the allospecific response on day 6 post-transplant, and we did not specifically assess the role of endogenous memory CD8^+^ T cells in this model. However, our antigen specific mouse model may be unsuitable to assess cell populations like endogenous CD8^+^ memory T cells, as the impact of such cells may be inherently altered, or possibly masked, in the face of a substantial antigen-specific immune response. The relationship between IRI-induced endogenous memory CD8^+^ T cell infiltration and the subsequent CD4^+^ alloantigen specific response, both of which result in graft infiltration with IFNγ-producing T cells and CTLA4Ig resistant rejection, will be an important future topic for investigation.

Following ischemia, a marked increase in the level of intra-graft IL-6 was observed in the early post-transplant period, prior to the appearance of significant lymphocytic infiltration. On staining, CD4^+^ and CD8^+^ were not seen at 1 day post-transplant, suggesting APC, including allograft resident DCs, as a potential source of IL-6. DCs are highly specialized to absorb oxidative stress signals and increase alloimmune activation via multiple routes including enhanced expression of MHC class II and positive costimulatory molecules, and the secretion of inflammatory cytokines^19^. Our group has previously shown that ischemic dendritic cells increase IL-6 production via activation of autophagy pathways^48^ and ischemic allografts from mice lacking autophagy-related gene-5 in DCs had a marked reduction in IL-6 expression^48^. Furthermore, *in vitro*, IL-6 production by ischemic DCs leads to increased proliferation and pro-inflammatory cytokine production by allospecific CD4^+^ T cells^16,18^. In our current study, depletion of APC, of which allograft resident DCs are a significant population^29^, led to a notable suppression of the early augmentation in intra-graft IL-6, again indicating that APCs are amongst the main producers of IL-6 within the organ. Depletion was also associated with a marked reduction in alloreactive T cell recruitment and subsequent graft rejection.

Despite elevated levels of IL-6, the predominant intra-graft T cell population following IRI was CD8^+^ IFNγ^+^ T cells. IL-6 is classically described to promote Th17 differentiation and oppose Th1 differentiation via upregulation of SOCS1, which inhibits IFNγ signaling during T cell activation^49,50^. However, IL-6 is a pleiotropic cytokine and under certain conditions, IL-6 may augment established Th1 responses, including Th1 mediated colitis and Th1 and Th17 immune reactivity in experimental autoimmune encephalitis^51–53^.

While the effect of IL-6 on Th differentiation could be model dependent, graft derived IL-6 has been previously described to be a potent driver of acute allograft rejection. IL-6^−/−^ cardiac allografts have significantly prolonged survival when transplanted into WT mice. The absence or blockade of IL-6 was associated with a significant reduction in chronic rejection and a decreased CD8^+^ IFNγ^+^ allospecific response, a similar effect to that observed response in our model following IRI^20,21^.

Migration of CD8^+^ T cells to the allograft is driven by antigen presentation by graft resident APCs or endothelial cells^54^. Elevated IL-6 levels following IRI could promote this process by augmenting the capacity of APCs to present antigen by mechanisms such as upregulating MHC class II and costimulatory molecules, further augmenting innate immune responses, and dampening the suppressive activities of Tregs^18,55,56^. Tregs have been shown to preferentially aggregate around DCs and inhibit their maturation^55^. However, donor-derived IL-6 is known to decrease intra-graft Treg numbers^57^ and in our model, IRI led to a reduction in allograft infiltration by regulatory T cells. This could also have led to enhanced DC activation and antigen presentation, promoting T cell infiltration.

In addition, IL-6 promotes retention of infiltrating inflammatory cells, through increased levels of CCL4, CCL5, RANTES and IP10^58^, and enhances pro-inflammatory T cell differentiation^21,53,59,60^. Indeed, in this model, blockade of IL-6 led to significantly decreased allograft infiltration with CD4^+^ and CD8^+^ T cells, with reductions in pro-inflammatory CD4^+^ IL17^+^ and CD4^+^ IFNγ^+^ T cells, while CD4^+^ Foxp3^+^ T cells were increased compared to untreated ischemic controls. These changes emphasize the effect of IL-6 in driving a pro-inflammatory T cell response to IRI and demonstrate the efficacy of IL-6 blockade in abrogating this response.

IL-6 also promotes endothelial cell ICAM-1 expression and chemokine production, promoting leukocyte recruitment to sites of inflammation^61^. Endothelial cells do not express the IL-6 receptor but do express gp130 and can respond to IL-6 trans-signaling, i.e. signaling mediated by IL-6/soluble IL-6 receptor (sIL6R) complex; leading to upregulation of ICAM-1^61–63^. Increased sIL6R levels can occur via differential mRNA splicing or cellular shedding, and is observed in several inflammatory conditions^64,65^. Whether increased sIL6R is present following IRI and could be a potential mechanism of endothelial cell activation and T cell recruitment to the ischemic allograft is an interesting question, worthy of future study.

Anti-IL-6 therapy has been proven to be clinically efficacious in treating rheumatoid arthritis and has been studied for treatment of graft versus host disease, among other applications^66–68^. CTLA4Ig has emerged as a promising therapeutic agent in transplantation, with a better safety profile than calcineurin inhibitors^69–71^. However, CTLA4Ig treatment has been complicated by an increased rate of early acute rejection episodes^69,71^ and increased alloimmune activation of ischemic organs could increase the risk of rejection in CTLA4Ig treated patients. While we have previously shown the utility of anti-IL-6 in a class II mismatch model, here, we used a more clinically relevant, full MHC mismatch model of prolonged ischemia, where we investigated the synergistic effects of anti-IL-6 with CTLA4Ig^72^. Our data indicate a significant decrease in heart graft survival following severe IRI in sCTLA4Ig treated recipients, and adding anti-IL-6 abrogates this effect, by alleviating inflammation, decreasing immune cell trafficking to the ischemic organ and decreasing vascular injury; resulting in similar graft survival as control non-ischemic grafts.

In summary, our current study indicates that CD4^+^ alloreactive T cells play a critical role in augmenting alloimmune responses following IRI. Our data suggests a model whereby IRI leads to increased IL-6 production by APC, allospecific CD4^+^ infiltration, leading to graft infiltration by CD8^+^ IFNγ^+^ T cells, resulting in enhanced allograft injury and poorer long-term graft outcomes. IL-6 blockade abrogates IRI-augmented alloimmunity and combination therapy with CTLA4Ig and anti-IL-6 could be a promising therapeutic strategy for patients who receive highly ischemic organs.

## Materials and Methods

### Mice

All animal experiments and methods were performed in accordance with the relevant guidelines and regulations approved by the Institutional Animal Care and Use Committee of Brigham and Women’s Hospital, Harvard Medical University, Boston, MA (protocol number: 2016N000167/04977).

C57BL/6, BALB/c, C57BL/6-Tg (TcraTcrb)1100Mjb/ (OTI), C57BL/6-Tg(TcraTcrb)425Cbn (OTII), and C57BL/6-Tg(CAG-OVA)916Jen/J mice were purchased from Jackson Laboratory (Bar Harbor, ME, USA) and used at 6–10 weeks of age. OTI and OTII mice are TCR transgenic mice specific to OVA peptide, which recognize either OVA_257–264_ or OVA _323–339_ in context of MHC class I or II, respectively.

### Heterotopic cardiac transplantation

Heterotopic intra-abdominal cardiac transplantation was performed using microsurgical techniques as described by Corry *et al*.^73^. For most experiments, donor hearts were harvested, and kept at 4 degrees Celsius in University of Washington (UW) solution for 8 hours prior to transplantation for ischemic conditions, whereas control group were transplanted within 30 minutes after harvest. For severely ischemic conditions (Fig. 6), hearts were stored in the above conditions for 16 hours prior to transplantation. Graft heartbeat was evaluated daily by palpation. Rejection was determined as very weak beating in OVA/OTII heart transplant model or complete cessation of cardiac contractility.

### *In vivo* treatment protocols

To deplete CD4^+^ T cells, recipient mice were treated intravenously with 1 mg of anti-CD4 antibody (clone GK1.5; Bio X Cell, West Lebanon, NH) on days −3, −2 and −1 before transplantation. CD4^+^ T cell depletion (≥98%) was confirmed in peripheral blood by flow cytometry. To deplete APCs, donor mice were injected with 0.5 mg liposomal clodronate (Encapsula NanoSciences, Nashville, TN) intraperitoneally on days −8, −5 and −1 before transplant as previously described^29^. For systemic IL-6 blockade, 0.1 mg anti-mouse IL-6 antibody (clone cMR16–1; Courtesy of Genentech) was injected intraperitoneally into allograft recipients on days 0 to 3 and then on alternate days until day 13. A single dose of CTLA4Ig 250 microgram was injected intraperitoneally on day two following transplantation.

### Lymphocyte extraction from transplanted heart grafts

Heart grafts were procured and flushed with PBS to remove any remaining clot. They were then minced in RPMI-1640 medium containing % 0.1 collagenase and incubated for one hour at 37 °C with 5% CO_2_ followed by addition of 0.1 M EDTA in PBS. After 5 minutes more incubation at 37 °C, 5 mM EDTA in PBS with 1% of FBS buffer was added. Cells were then filtered through 70 um strainer, counted and stained for flow cytometry.

### Flow cytometry

Flow cytometric analysis of graft infiltrating cells, draining lymph node (DLN) and spleen was performed and each leukocyte population was quantified. For intracellular cytokine staining, cells were stimulated *ex-vivo* with PMA (50 ng/ml) and Ionomycin (500 ng/ml) in combination with GolgiStop for 4 hours, then permeabilized and stained with fluorochrome conjugated antibodies against IFNγ and IL-17. All antibodies were purchased from BD (Becton Dickinson Franklin Lakes, NJ). Cells were run on FACSCanto II (BD Biosciences, Franklin Lakes, NJ) instrument. Data were analyzed using FlowJo software.

### ELISPOT assay

To assess production of murine IFNγ, ELISPOT assay was performed according to the manufacturer’s instructions (BD Biosciences). Briefly, immunospot plates (Millipore) were coated with IFNγ primary antibody overnight. Donor splenocytes were harvested, processed and irradiated at 3000 Rads and then plated with recipients’ whole splenocytes in a 1:1 ratio and incubated in 37  °C for 24 hours. Cells were then washed out and detection antibody was added and incubated overnight. After development with the chromogen, the total number of spots per well were quantified using an ImmunoSpot Analyzer (Cellular Technology, Cleveland, OH, USA).

### Immunohistochemistry

Heart grafts harvested at certain time points post-transplant were fixed in formalin and embedded to paraffin block. Samples were then cut into 5 micron thick and stained with Hematoxylin and Eosin stain (H&E), CD4 and CD8. Anti-CD4 and CD8 antibodies were purchased from BD Biosciences.

### Quantitative RT-PCR

Harvested cardiac grafts were flash frozen in liquid nitrogen and used for RNA extraction by SYBR Green-based detection. Tissues were homogenized and sonicated, and RNA was isolated using RNeasy mini kit (Qiagen, Valencia, CA). Harvested RNA was subsequently measured and used to prepare cDNA using iScript cDNA synthesis kit (Bio-Rad laboratories, Inc., Hercules, CA). Quantitative PCR was then performed for IL-6 genes. The primer for IL-6 is; the forward 5′-CCGGAGAGGAGACTTCACAG-3′ and the reverse 5′-GGAAATTGGGGTAGGAAGGA-3′. Expression was assessed as compared to GAPDH expression. Relative gene expression was calculated using the 2^ΔΔCT^ method.

### Statistics

Kaplan-Meier survival graphs were constructed and log rank comparison of the groups was used to calculate *p*-values for survival comparisons between various groups. Data analysis was performed using GraphPad Prism (GraphPad Software, Inc., San Diego, CA). Differences between groups for cellular infiltration, luminex and ELISPOT numbers were evaluated by student’s *t*-test to determine significance. *p < 0.05 was considered a significant difference.

### Data Availability

All data generated or analyzed during this study are available from the corresponding author on reasonable request.

## Electronic supplementary material

Supplementary material
