# Supplementary material for: Ischemia augments alloimmune injury through IL-6-driven CD4+ alloreactivity
Source: Sci Rep. 2018 Feb 6;8:2461. doi: 10.1038/s41598-018-20858-4 (PMC5802749; doi:10.1038/s41598-018-20858-4)
Supplement: Supplementary file 1 — Supplementary material [file 41598_2018_20858_MOESM1_ESM.pdf]

Ischemia augments alloimmune injury through IL-6-driven CD4$^+$ alloreactivity

Mayuko Uehara[1‡], Zhabiz Solhjou[1‡], Naima Banouni[1], Vivek Kasinath[1], Ye Xiaqun[1], Li Dai[1], Osman Yilmam[1], Mine Yilmaz[1], Takaharu Ichimura[2], Paolo Fiorina[3], Paulo N. Martins[4], Shunsuke Ohori[1], Indira Guleria[1], Omar H. Maarouf[1], Stefan G. Tullius[5], Martina M. McGrath[1]*, Reza Abdi[1]*

1 Transplantation Research Center, Renal Division, Brigham and Women's Hospital, Harvard Medical School, Boston, MA, USA

2 Renal Division, Brigham and Women's Hospital, Harvard Medical School, Boston, MA, USA

3 Division of Nephrology, Boston Children Hospital, Harvard Medical School, Boston, MA, USA

4 Division of Surgery, University of Massachusetts Medical School, Boston, MA, USA

5 Division of Transplant Surgery and Transplantation Surgery Research Laboratory, Brigham and Women's Hospital, Harvard Medical School, Boston, MA, USA

‡ These authors contributed equally to this work.

*Address correspondence to:

Reza Abdi, MD

Transplantation Research Center, Brigham and Women's Hospital

221 Longwood Ave, Boston MA 02115, USA

Tel: 617-732-5259, Fax: 617-732-5254, E-mail: rabdi@rics.bwh.harvard.edu

Martina M. McGrath

Transplantation Research Center, Brigham and Women's Hospital

221 Longwood Ave, Boston MA 02115, USA

Tel: 617-732-5259, Fax: 617-732-5254, E-mail: MMCGRATH8@BWH.HARVARD.EDU

Supplementary Figure Legend:

Supplementary Figure 1. Allospecific $CD8^+$ T cells lead to prompt rejection, which is not augmented by IRI. Control or ischemic OVA hearts were transplanted into OTI recipients. (A) Heart grafts were rejected around 3 days post-transplant with no difference between control and ischemic groups (MST: 3 vs. 3 days, p=0.51, n=5/group). (B) Heart grafts were harvested at 4 days post-transplant for histological analysis. Both control and ischemic grafts showed severe allograft injury with dense lymphocyte infiltration, parenchymal necrosis and hemorrhage in both groups. (Scale bar 100µm for H&E. Inset scale bar 30µm).

Supplementary Figure 2. Analysis of graft infiltrating $CD4^+$ T cell populations shows a pro-inflammatory phenotype following IRI. Control or ischemic OVA hearts were transplanted into OT II recipients and harvested at day 6 post-transplant. Graft infiltrating lymphocytes were isolated and underwent intracellular staining to analyze intra-graft $CD4^+$ T cell populations. (A) No differences were observed in numbers of graft-infiltrating $CD4^+$ $Foxp3^+$ T cells between ischemic and control grafts (absolute cell count: 6498±844.7 vs. 5364±2925.1, p=0.6, n=3/group). (B) Absolute number of $CD4^+$ $IL17^+$ cells were higher in ischemic grafts compared to control (absolute cell count: 2615±461.5 vs. 816.1±328.9, *p<0.05, n=3/group). (C) A greater number of $CD4^+$ $IFN\gamma^+$ cells were found in ischemia group compared to control (absolute cell count: 8518±1503 vs. 2206±888.8, *p<0.05, n=3/group). Representative flow plots for all are shown.

Supplementary Figure 3. IRI did not alter frequency of pro-inflammatory T cells in secondary lymphoid tissues. Control or ischemic OVA hearts were transplanted into OTII

recipients and spleen and graft draining lymph nodes were harvested at day 6 post-transplant. (A) No differences were seen in frequency of splenic $CD4^+$ $Foxp3^+$ or $CD8^+$ $IFN\gamma^+$ T cell between groups, as assessed by flow cytometry. (B) No differences were seen in frequency of $CD4^+$ $IL17^+$ T cell or $CD8^+$ $IFN\gamma^+$ T cell in DLN between both groups by flow cytometry.

Supplementary Figure 4. IRI leads to increased allospecific IFNγ production by splenocytes; an effect abrogated by $CD4^+$ T cell depletion. (A) Control or ischemic OVA hearts were transplanted into OTII recipients, and spleens were harvested at day 6 post-transplant. Recipient whole splenocytes were incubated with irradiated OVA cells and allospecific IFNγ production was measured by ELISPOT analysis. Significantly greater splenocyte IFNγ production was seen in ischemia group (68.17±3.32 vs. 25.63±3.45 spots/10^6 cells, ***p<0.001, n=3/group). (B) OTII recipients were depleted of $CD4^+$ T cell before transplantation of control or ischemic OVA heart grafts. Spleens were harvested at 6 days post-transplant and allospecific IFNγ production was assessed by ELISPOT. No difference was seen in splenocyte IFNγ production in control or ischemia group following CD4 depletion (2.4±0.5 vs. 9.3±3.7 spots/10^6 cells, p=0.13, n=3/group).

Supplementary Figure 5. Liposomal clodronate depletes intra-graft CD11c and F4/80 cells. (A) Flow cytometric analysis of cardiac APCs in donor heart after clodronate administration showed significant reduction in $CD11c^+$ DCs (control vs. clodronate treatment; $109.9\times10^3 \pm 13.0\times10^3$ vs. $29.2\times10^3 \pm 8.4\times10^3$, *$p$<0.05, n=3/group). (B) A non-significant decrease in the absolute number of $F4/80^+$ macrophages was seen after clodronate administration (control vs. clodronate treatment; $545.1\times10^3 \pm 223.5\times10^3$ vs. $318.1\times10^3 \pm 74.7\times10^3$, p=0.39, n=3/group).

Supplementary Figure 6. Allograft resident APCs are the main source of increased IL-6 within ischemic allografts. (A) Ischemic and control OVA hearts were transplanted into OTII recipients, harvested on day 1, 3 and 6 post-transplant and RNA was extracted for PCR analysis at each time point. IL-6 gene expression was 8 times higher in ischemic grafts than control at 1 day post-transplant, and remained significantly higher up to day 3. (*$p<0.05$ for day 1, n=4/group, and *$p<0.05$ for day 3, n=3/group, respectively). (B) Control and ischemic grafts were harvested at day 1 and stained by immunohistochemistry for analysis of graft infiltrating cells. At day 1, no infiltrating cells, $CD4^+$ and $CD8^+$ T cells were detected in either control or ischemic grafts. (Scale bar 100μm for H&E, CD4 and CD8 stain. Inset scale bar 30μm)**.** (C) Control, ischemic or APC depleted ischemic OVA hearts were harvested at day 1 post-transplant from OTII recipients. Following RNA extraction, PCR analysis demonstrated a significant reduction of IL-6 expression, with undetectable IL-6 expression in APC depleted ischemic grafts as compared to non-depleted ischemic grafts.

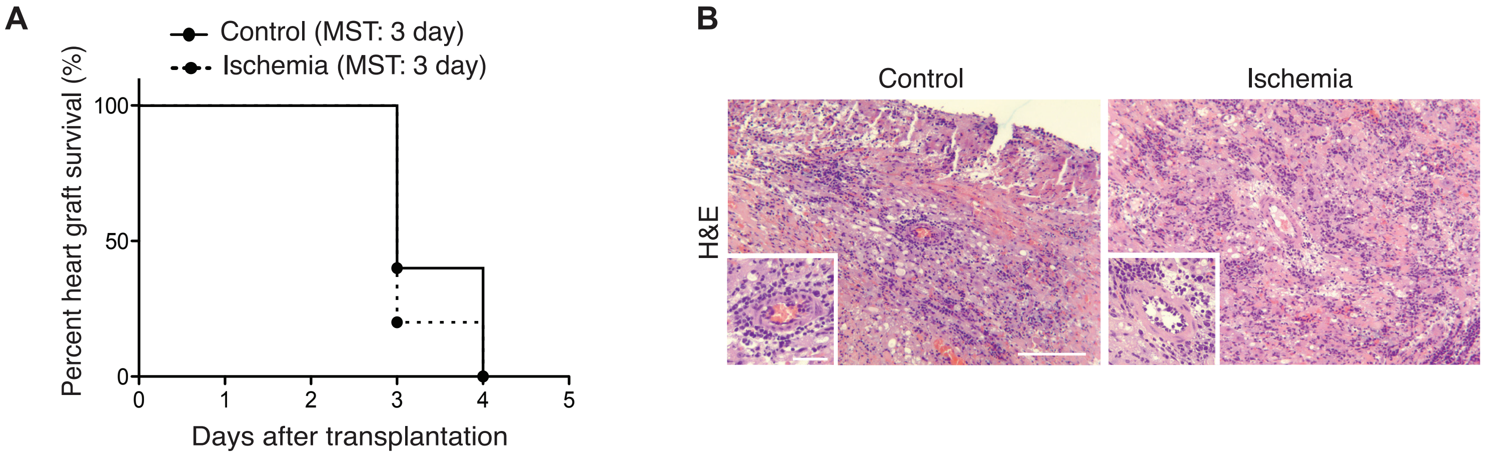

Uehara et al. Suppremenary Figure 1

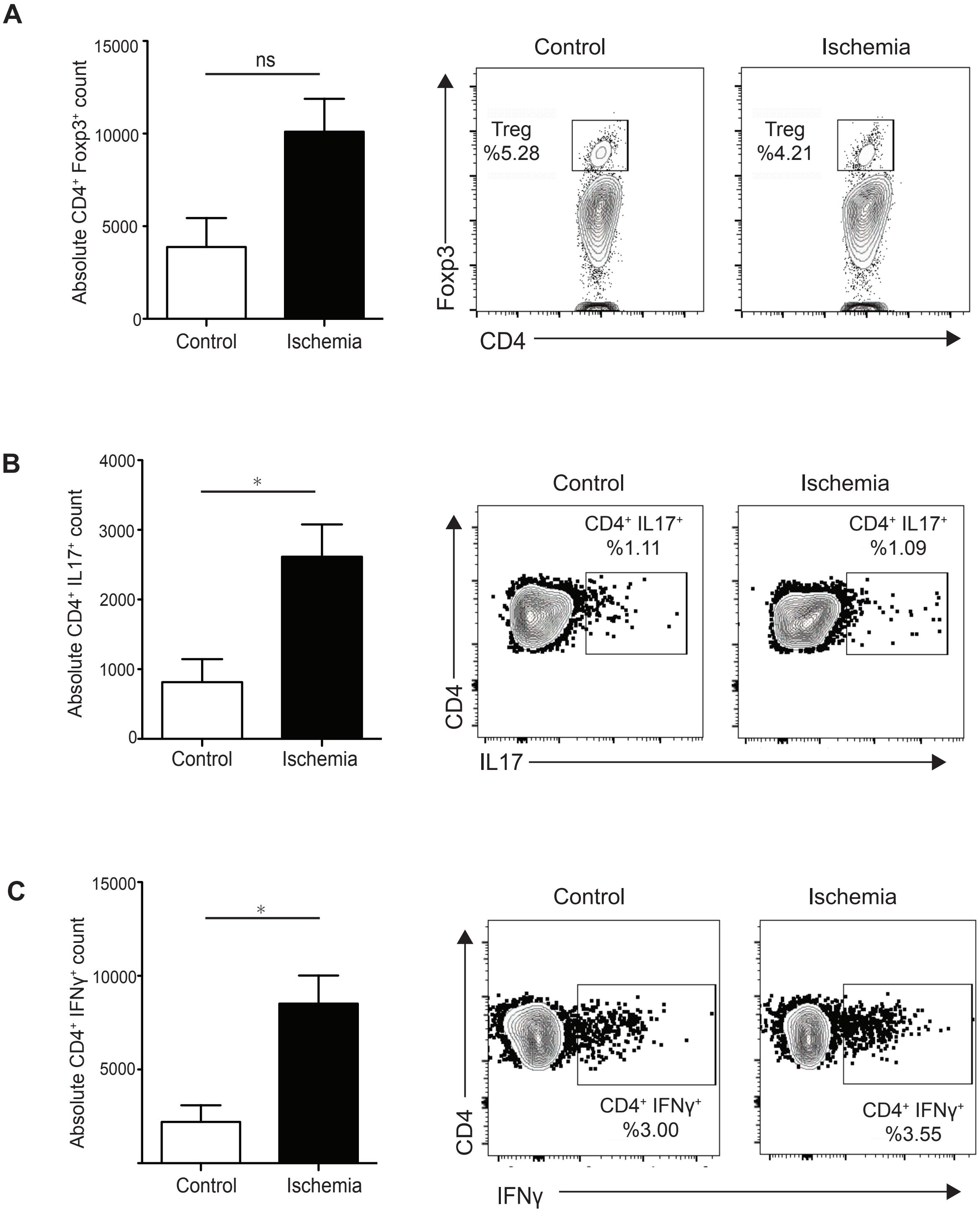

Uehara et al. Supplementary Figure 2

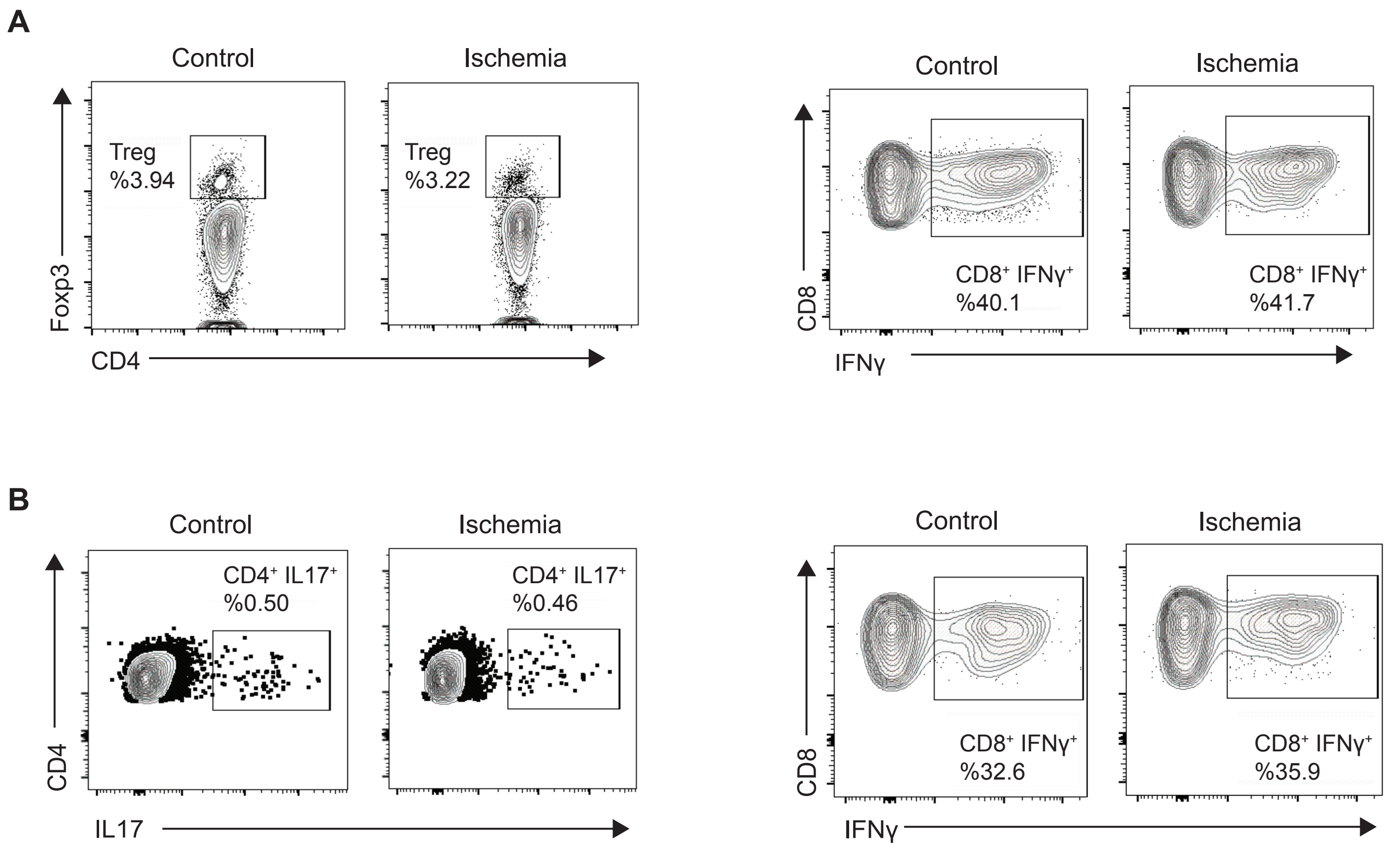

Uehara et al. Supplementary Figure 3

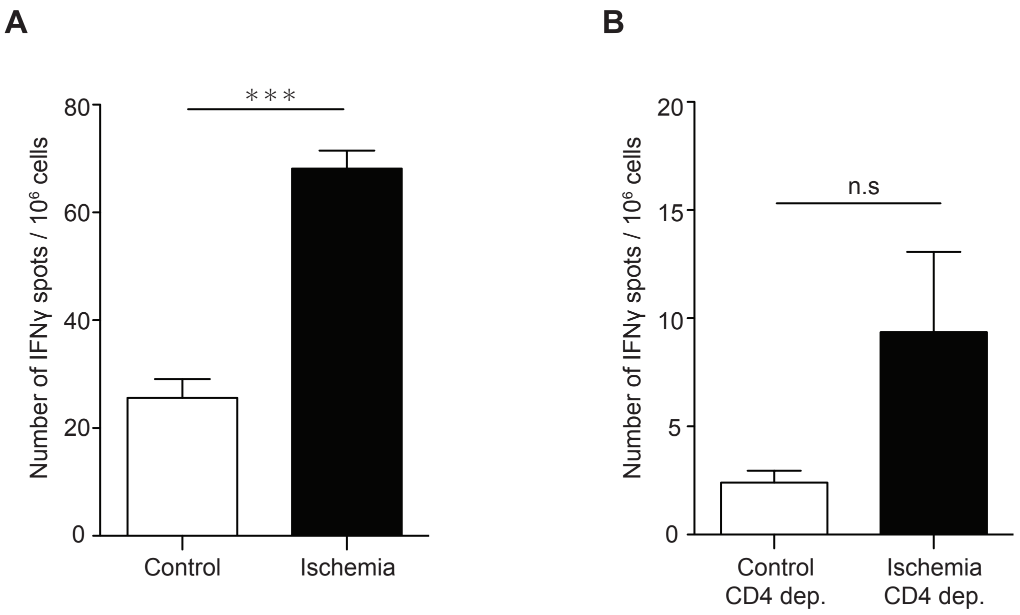

Uehara et al. Supplementary Figure 4

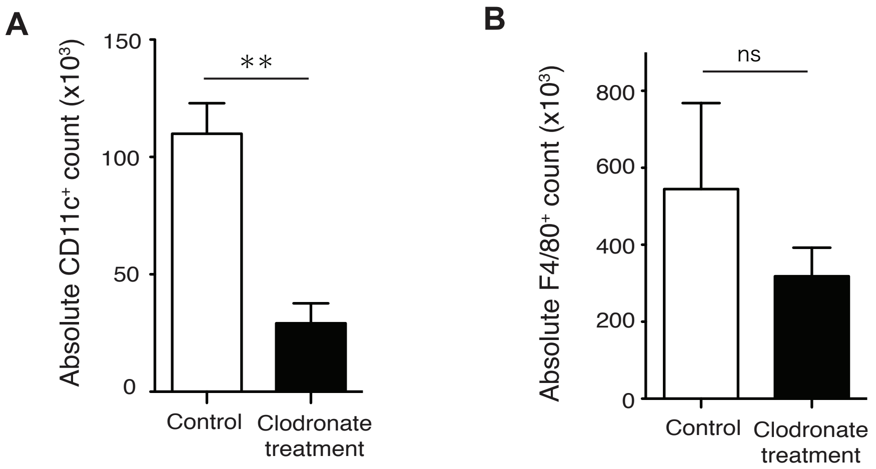
A
150
100
50
0
Absolute CD11c+ count (x10³)
**
Control
Clodronate treatment
B
800
600
400
200
0
Absolute F4/80+ count (x10³)
ns
Control
Clodronate treatment

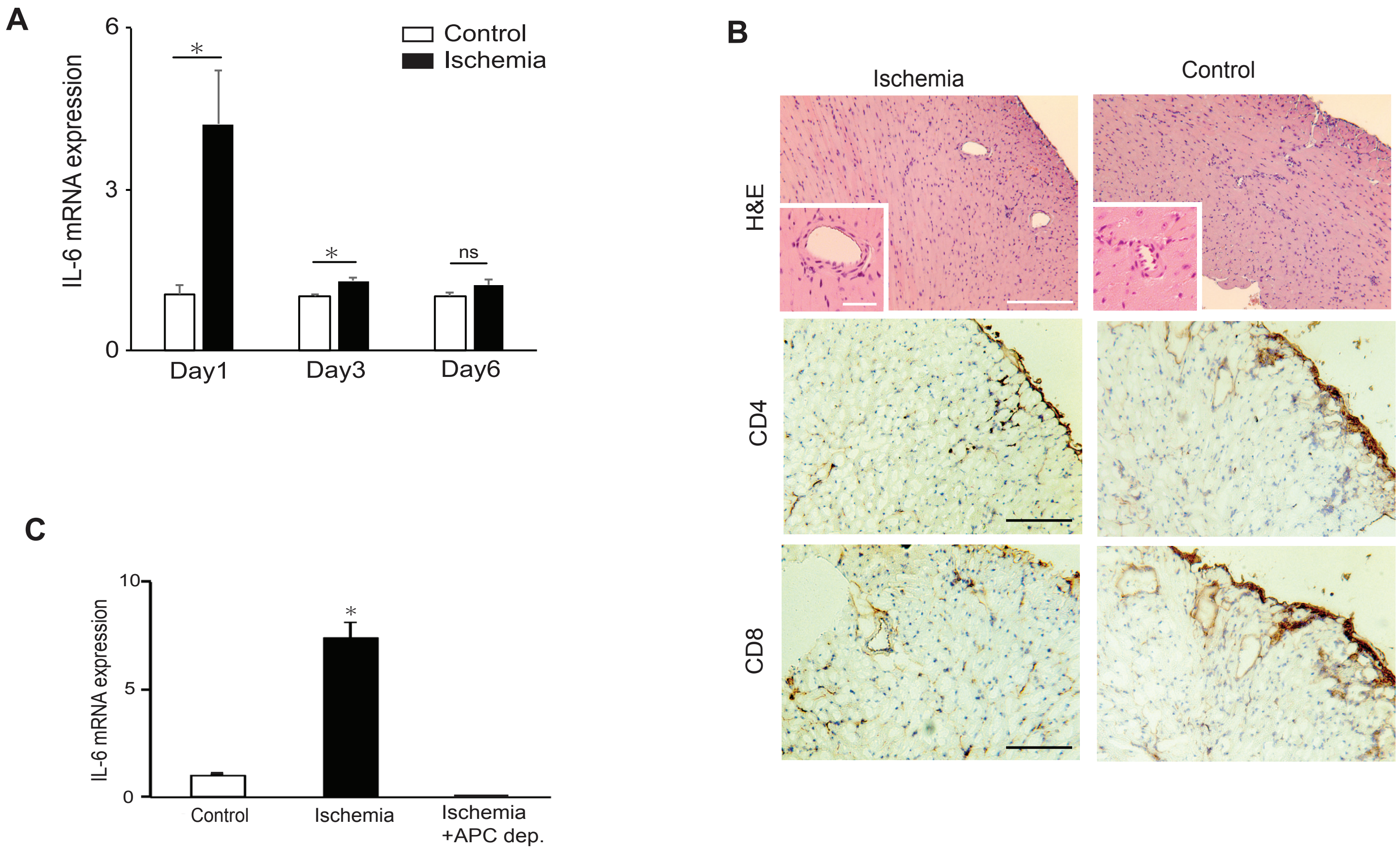

Uehara et at. Supplementary Figure 6
